# *Oesophagostomum dentatum* Extract Modulates T Cell-Dependent Immune Responses to Bystander Antigens and Prevents the Development of Allergy in Mice

**DOI:** 10.1371/journal.pone.0067544

**Published:** 2013-07-02

**Authors:** Irma Schabussova, Onisa Ul-Haq, Elisabeth Hoflehner, Johnnie Akgün, Angelika Wagner, Gerhard Loupal, Anja Joachim, Bärbel Ruttkowski, Rick M. Maizels, Ursula Wiedermann

**Affiliations:** 1 Institute of Specific Prophylaxis and Tropical Medicine, Center for Pathophysiology, Infectiology and Immunology, Medical University of Vienna, Vienna, Austria; 2 Department of Pathobiology, Institute of Pathology and Forensic Veterinary Medicine, University of Veterinary Medicine, Vienna, Austria; 3 Institute of Parasitology, University of Veterinary Medicine, Vienna, Austria; 4 Institute of Immunology and Infection Research, University of Edinburgh, Edinburgh, United Kingdom; Université Paris Descartes, France

## Abstract

One third of the human population is currently infected by one or more species of parasitic helminths. Certain helminths establish long-term chronic infections resulting in a modulation of the host’s immune system with attenuated responsiveness to “bystander” antigens such as allergens or vaccines. In this study we investigated whether parasite-derived products suppress the development of allergic inflammation in a mouse model. We show that extract derived from adult male *Oesophagostomum dentatum* (eMOD) induced Th2 and regulatory responses in BALB/c mice. Stimulation of bone marrow-derived dendritic cells induced production of regulatory cytokines IL-10 and TGF-beta. In a mouse model of birch pollen allergy, co-administration of eMOD with sensitizing allergen Bet v 1 markedly reduced the production of allergen-specific antibodies in serum as well as IgE-dependent basophil degranulation. Furthermore, eMOD prevented the development of airway inflammation, as demonstrated by attenuation of bronchoalveolar lavages eosinophil influx, peribronchial inflammatory infiltrate, and mucus secretion in lungs and IL-4 and IL-5 levels in lung cell cultures. Reduced secretion of Th2-related cytokines by birch pollen-re-stimulated splenocytes and mesenteric lymph node cells was observed in eMOD-treated/sensitized and challenged mice in comparison to sensitized and challenged controls. The suppressive effects of eMOD were heat-stable. Immunization with model antigens in the presence of eMOD reduced production of antibodies to thymus-dependent but not to thymus-independent antigen, suggesting that suppression of the immune responses by eMOD was mediated by interference with antigen presenting cell or T helper cell function but did not directly suppress B cell function. In conclusion, we have shown that eMOD possesses immunomodulatory properties and that heat-stable factors in eMOD are responsible for the dramatic suppression of allergic responses in a mouse model of type I allergy. The identification and characterization of parasite-derived immune-modulating molecules might have potential for designing novel prophylactic/therapeutic strategies for immune-mediated diseases.

## Introduction

Infections with helminth parasites represent a global health problem with more than one billion people infected worldwide. Exposure to helminth parasites has a major impact on the development and reactivity of the host’s immune system. In order to prevent their expulsion or reduce severe pathology, helminth parasites engage complex mechanisms to “act the innocent” in order to avoid attention and/or to actively manipulate the effector mechanism of the host immune system [1].

Even though they have common immunological features (elevated levels of IgE, eosinophilia, and production of Th2 cytokines such as IL-4 and IL-5), epidemiological studies revealed an inverse relationship between helminth infections and allergic diseases. For example, infections with *Schistosoma haematobium* or hookworms were associated with protection from atopic reactivity [2], [3]. Similarly, infection with *A. lumbricoides* or *Trichuris trichiura* was associated with lower prevalence of skin prick test reactivity [4], [5]. Studies reporting a significant increase in allergen skin sensitization following anthelmintic treatment provide additional evidence that helminth infection and allergic sensitization are likely to be interrelated. [6], [7], [8], [9]. Such observations recently received considerable interest, leading to intervention studies using worms as a therapy of immunological disorders. Due to the fact that *T. suis*, the pig whipworm, is likely to be non-pathogenic in human subjects, the majority of reported clinical trials have been performed with this parasite.

Notably, earlier clinical studies provided encouraging results on the beneficial effect of application of *T. sui*s eggs to patients with multiple sclerosis [10] as well as to patients with Th1-mediated Crohn’s disease or Th2-mediated ulcerative colitis [11], [12], [13]. However, no beneficial effect of experimental *T. suis* infection was found in two clinical trials in allergic rhinitis [14], [15]. Similarly, experimental infection with the hookworm *Necator americanus* did not result in clinically significant improvement of airway responsiveness [16], [17] but induced regulatory responses in celiac individuals [18]. While clinical studies performed thus far have demonstrated the safety and provided evidence that controlled infections with helminth parasites are well tolerated, greater consistency and wider application may be achieved by identifying active parasite-derived substances which do not require live infections of patients with helminth parasites.

Several parasite-derived products have revealed their potential to inhibit immunopathology in various animal models. For example, soluble products from *T. suis*, *Trichinella spiralis* [19], or *S. japonicum* [20] suppressed clinical signs in murine experimental autoimmune encephalomyelitis. Moreover, mice treated with extracts of the tapeworm *Hymenolepis diminuta* were protected against colitis induced by DNBS [21]. Finally, excretory/secretory products derived from *Nippostrongylus brasiliensis* [22], *Heligmosomoides polygyrus* [23], *Toxascaris leonina* [24], or *S. mansoni* [25] suppressed allergic airway inflammation in mice.

*Oesophagostomum dentatum*, the nodule worm, is a gastrointestinal nematode parasite of pigs worldwide, which follows a direct transmission cycle. Ingested larvae invade the mucosa of cecum and colon, moult to L4 larvae and return to the intestinal lumen, where they mature. Adult male and female worms cause chronic patent infections without overt clinical signs [26].

In this study we investigated whether products derived from adult male *O. dentatum* (eMOD) modulate responses to sensitizing allergen in a mouse model of type I allergy. We found that co-administration of eMOD with the major birch pollen allergen Bet v 1 led to significant suppression of both humoral and cellular allergic responses, as well as airway eosinophilia. The allergy-protective effect of eMOD is mediated by heat-stable component, interfering possibly with antigen presenting cell or T cell function.

## Materials and Methods

### Animals

6–8 week-old female BALB/c mice were obtained from Charles River (Sulzfeld, Germany) and maintained under conventional housing conditions. Pigs were kept at the animal facilities of the Institute of Parasitology, University of Veterinary Medicine, Vienna. All experimental protocols were reviewed and approved by the Austrian Federal Ministry of Science and Research.

### Preparation of Parasite Extract

*Oesophagostomum dentatum*, OD-Hann (Joachim et al. 1997) is routinely maintained by infection of parasite-free pigs. Adult male worms were isolated from the intestines of pigs during the patent stage of infection according to Slotved *et al.* [27]. Whole body extract from male worms (eMOD) was prepared by homogenization of worms isolated from the intestine of pigs during the patent stage of infection. Worms were extensively washed in PBS, snap-frozen in liquid nitrogen and homogenized mechanically in PBS, followed by centrifugation at 10,000×*g* for 15 min at 4°C. The supernatant was passed through a 0.22 µm filter and the total protein concentration was quantified by the bicinchoninic acid assay (BCA Protein Assay; Thermo Scientific) according to the manufacturer’s protocol. Extracts were tested in the Limulus Amoebocyte Lysate (LAL) assay (Endpoint Chromogenic LAL Assays; Lonza). The levels of endotoxin were below 0.1 EU in 1 µg of extract. Samples were stored at −80°C until required. Heat treatment was performed by incubation at 96°C for 15 min.

### Generation and Stimulation of Bone Marrow Derived Dendritic Cells

Bone marrow derived dendritic cells (BM-DC) were generated as previously described [28]. Briefly, the bone marrow precursors were isolated from femurs and tibias of BALB/c mice. Cells were cultured at 2×10^5^/ml in bacteriological Petri dishes in 10 ml culture medium with GM-CSF (20 ng/ml; Sigma-Aldrich). Fresh medium was added at days 3 and 6 and BM-DC were used on day 8 of culture. BM-DC (10^6^ cells/ml) were stimulated with eMOD at a final concentration of 100 µg/ml or left untreated for 24 h, after which supernatants were tested by ELISA.

### Immunization

Mice were immunized by i.p. injections of 50 µg aluminium-precipitated eMOD (eMOD group) or sham treated with PBS-alum (sham group) on days 0 and 10. Blood, spleen and mesenteric lymph nodes (MLN) were collected on sacrifice (day 21). In order to investigate the impact of eMOD on third-party antigens, mice were immunized i.p. with 50 µg of eMOD (eMOD group); 10 µg of diphtheria toxoid-alum (DT group); 50 µg of eMOD plus 10 µg of diphtheria toxoid (eMOD/DT group); 200 µg of NIP-Ficoll (NIP group) or 50 µg of eMOD plus 200 µg of NIP-Ficoll (eMOD/NIP group) on days 0 and 7. Blood was collected on day 21. Blood samples were allowed to coagulate at room temperature for at least 4 h and centrifuged for 10 min at 1,500×*g*. Serum was collected and stored at −20°C for further analysis. Single-cell suspensions were prepared from spleens, lungs and MLN. Cells (2.5×10^7^/ml) were stimulated with eMOD (100 µg/ml) or media alone in 96 well-plates at 37°C for 72 h in culture medium (RPMI 1640 supplemented with 10% heat-treated FCS, 2 mM L-glutamine, 100 U/ml penicillin, 100 µg/ml streptomycin). Cell-free supernatants were stored at −20°C for further analysis.

### Mouse Model of Birch Pollen Induced Allergic Airway Inflammation

Induction of allergic airway inflammation was performed as previously described [29]. Briefly, mice were sensitized i.p. with three injections of 1 µg of recombinant Bet v 1 (Biomay, Vienna, Austria) precipitated with 100 µl of aluminium hydroxide (alum, Serva, Heidelberg, Germany) on days 0, 14, and 28; and then challenged intranasally with birch pollen extract (BP; Allergon, Välinge, Sweden; 100 µg/mouse in final volume of 30 µl) on three consecutive days (days 35, 36 and 37). Mice were terminally anesthetized 72 h after the final airway challenge (day 40).

### Bronchoalvelar Lavage and Differential Cell Counts

Bronchoalveolar lavage fluid (BALF) was collected by cannulating the trachea and injecting/recovering 2×1 ml PBS containing a protease inhibitor cocktail (Complete® Protease Inhibitor cocktail Tablets; Roche, Mannheim, Germany). BALF was centrifuged at 300×*g* at 4°C for 5 min and cell-free supernatants were stored at −20°C for further analysis. The cell pellet was resuspended in 200 µl of PBS; total leukocytes were counted and cytospins (4×10^4^ cells; Shandon Cytospin®, Shandon Southern Instruments, USA) were stained with haematoxylin and eosin (H&E Hemacolor®, Merck, Darmstadt, Germany) for differential cell counts (200 cells were counted per cytospin).

### Lung Histology

Following bronchoalveolar lavage, the lungs were fixed with 10% formaldehyde-PBS and paraffin-embedded. Tissue sections were stained with H&E. Airway mucus occlusion was analyzed on periodic acid-Schiff-stained (PAS, Sigma-Aldrich) sections.

### Immunoglobulin Levels in Blood Serum

Blood samples were taken by tail bleeding on the day of sacrifice and sera were stored at −20°C. Levels of antigen-specific serum antibodies were measured by ELISA. Microtitre plates (Nunc, Wiesbaden, Germany) were coated with eMOD (2 µg/ml), Bet v 1 (2 µg/ml), DT (10 µg/ml) or NIP-BSA (2 µg/ml) in coating buffer (0.1 M carbonate-bicarbonate buffer). Serum samples were diluted 1/100 for IgM and IgG, 1/1000 for IgG1, 1/500 for IgG2a, and 1/10 for IgE and IgA. Rat anti-mouse IgM, IgG, IgG1, IgG2a, IgE and IgA antibodies (1/500; Pharmingen, San Diego, CA) were applied and peroxidase-conjugated mouse anti-rat IgG antibodies (1/1000; Jackson, Immuno Labs., West Grove) were used for the detection. In order to measure specific IgE levels in sera, a rat basophil leukemia (RBL) cell mediator release assay was performed as previously described [30]. Briefly, RBL-2H3 cells were incubated with sera at a dilution of 1/100. Degranulation of RBL cells was induced by adding 0.3 µg of Bet v 1 diluted in 100 µl of Tyrode’s buffer. Data represent mean values ±SEM of percentages of total β-hexosaminidase activity after addition of 1% Triton X-100 and are shown after subtractions of baseline release levels obtained with pre-immune sera.

### Cytokine Measurements

Levels of IL-4, IL-5, IL-10, and TGF-β in supernatants were measured by ELISA (Endogen, Cambridge, MA) according to the manufacturer’s instructions.

### Statistical Analysis

All data are shown as mean ±SEM. Significance was analyzed using the non-parametric Mann-Whitney U-test (Graph Prism; Graph Pad Software, Inc, San Diego, CA). Differences were considered significant at p<0.05.

## Results

### Humoral and Cellular Responses induced by Immunization with Crude Extract of Male *Oesophagostomum dentatum*

**Figure 1:**
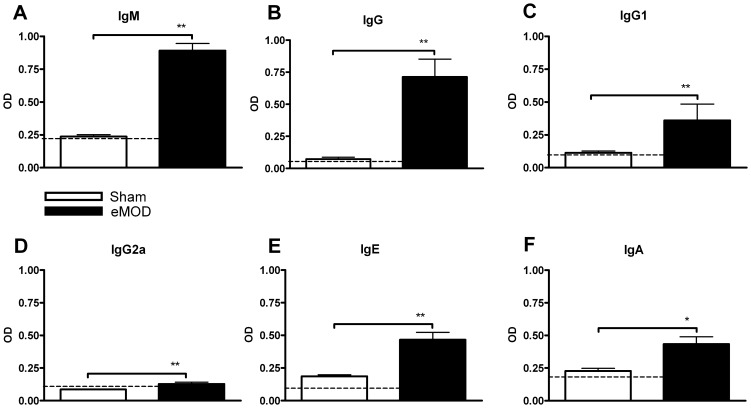
Antibody production in mice immunized with eMOD. Mice were immunized intraperitoneally with 50 µg of eMOD-alum (dark bars) or sham treated with PBS-alum (white bars) on days 0 and 10. Serum was collected on day 21. The levels of eMOD-specific IgM (A), IgG (B), IgG1 (C), IgG2a (D), IgE (E), and IgA (F) were detected by ELISA. The dashed line indicates the background level of the assay. Results are representative of three repeat experiments each with four to six mice per group. Data are expressed as mean ±SEM. *p<0.05; **p<0.01.

To analyze whether BALB/c mice can be primed *in vivo* to mount specific humoral and cellular immune responses to *O. dentatum*, mice were immunized twice, 10 days apart, with the crude extract of male *O. dentatum* (eMOD) in alum or with PBS in alum. At 11 days following the second immunization, *O. dentatum*-specific antibody production in serum and *O. dentatum*-specific cytokine production in eMOD re-stimulated spleen and MLN cell cultures were assessed. As shown in Fig. 1, by day 21 a substantial production of *O. dentatum*-specific antibodies was detected. Immunization with *O. dentatum* extract induced high levels of total IgG antibodies (Fig. 1 B) as well as specific IgG1 (Fig. 1 C). The production of anti-eMOD-IgG2a (Th1-associated isotype; Fig. 1 D) was significantly higher in *O. dentatum*-immunized mice compared to sham treated controls, even though the titres reached relatively low levels. Furthermore, immunization with *O. dentatum* induced high levels of specific IgM (Fig. 1 A), IgE (Fig. 1 E), and IgA (Fig. 1 F) antibodies in serum.

**Figure 2:**
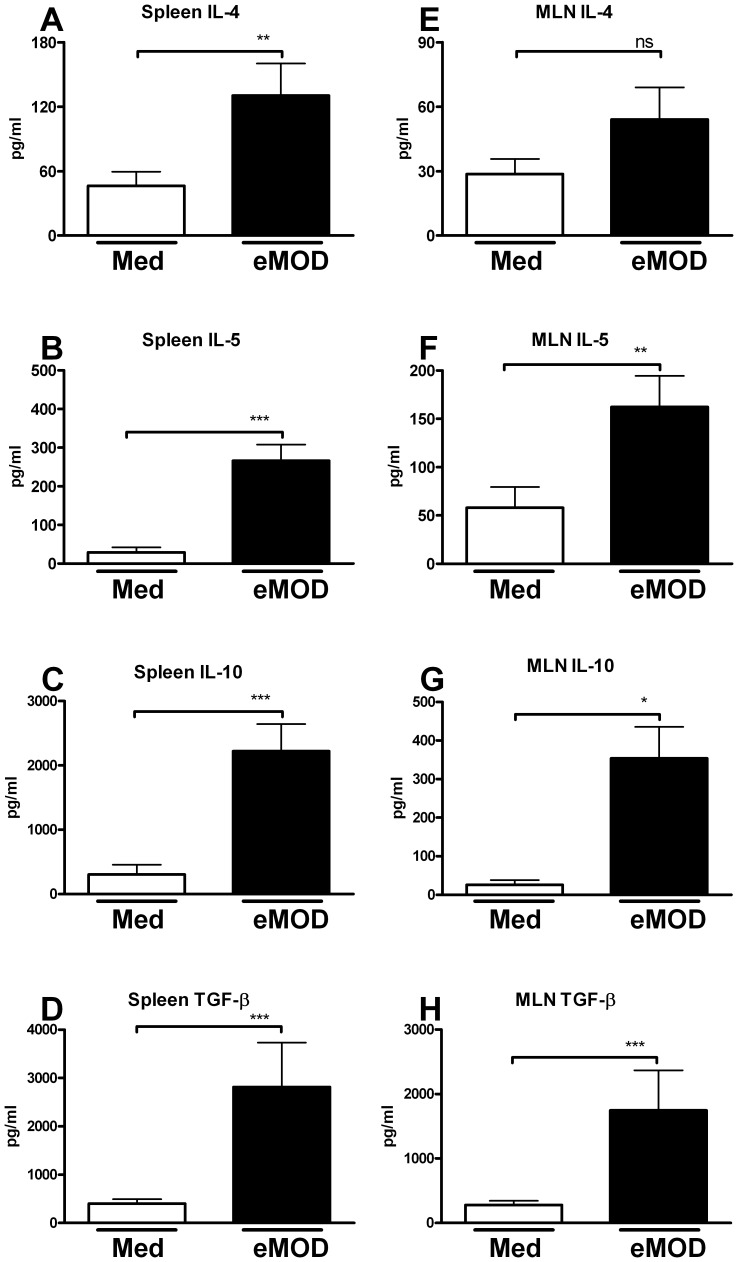
Immunization with eMOD induces Th2 and regulatory responses. Mice were immunized intraperitoneally with 50 µg of eMOD-alum on days 0 and 10. Spleen and MLN for cell preparation were collected on day 21. Cell cultures were stimulated with 100 µg of eMOD (dark bars; eMOD) or left untreated (white bars; Med) for 72 hours. Concentrations of IL-4 (A, E), IL-5 (B, F), IL-10 (C, G) and TGF-β (D, H) in the supernatants were measured by ELISA. Results represent three combined experiments each with four to six mice per group and are expressed as mean ±SEM. *p<0.05; **p<0.01; ***p<0.001.

The production of the Th2-associated cytokines IL-4 and IL-5 as well as of regulatory cytokines IL-10 and TGF-β were significantly increased in spleen cell cultures derived from eMOD-immunized mice stimulated with eMOD, in comparison to cells stimulated with medium alone (Fig. 2 A–D). Similar results were obtained for MLN cell cultures (Fig. 2 E–H). Furthermore, immunization with eMOD in the absence of alum triggered the production of specific IgG in serum and re-stimulation of spleen cell cultures with eMOD in these mice led to significant induction of IL-4, IL-5, IL-10 and TGF-β (data not shown). Levels of IFN-γ did not differ between eMOD-stimulated and untreated cultures (data not shown).

### Dendritic Cells Stimulated with eMOD Produce Regulatory Cytokines

**Figure 3:**
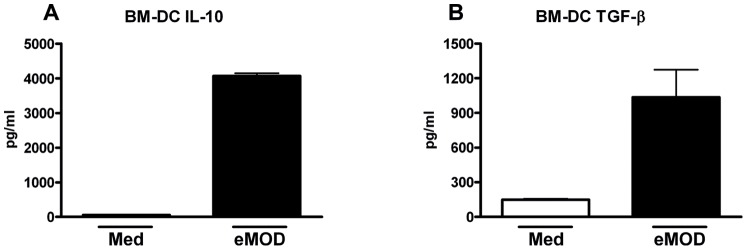
Stimulation of BM-DC with eMOD results in the production of regulatory cytokines. Bone marrow-derived dendritic cells (BM-DC) derived from naïve BALB/c mice were stimulated with 100 µg/ml of eMOD (dark bars; eMOD) or left untreated (white bars; Med) for 24 hours. Levels of IL-10 (A) and TGF-β (B) in the supernatants were measured by ELISA. Results are representative of three repeat experiments. Data are expressed as mean ±SEM.

Dendritic cells (DC) are professional antigen-presenting cells which are critical for initiating but also for regulating immune responses. In order to investigate whether DC are important players in induction of regulatory cytokines by eMOD, bone marrow-derived DC (BM-DC) from naïve BALB/c mice were stimulated with eMOD for 24 h. Cells stimulated with eMOD induced increased levels of IL-10 (up to 76-fold) and TGF-β (up to 6-fold) in comparison to cells stimulated with medium alone (Fig. 3). Levels of IL-12p40, IL-12p70 and TNF-α were not elevated in BM-DC cultures after stimulation with eMOD when compared to controls (data not shown).

### Application of eMOD during Sensitization Down-regulates Allergen-specific Antibody Production in a Mouse Model of Type I Allergy

**Figure 4:**
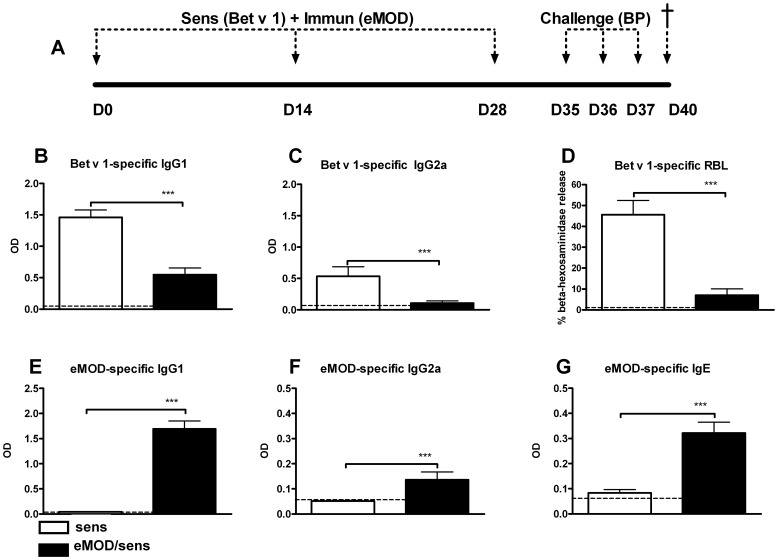
Co-application of eMOD at sensitization suppresses Bet v 1-specific but not eMOD-specific humoral responses. (A) A diagram of experimental setup. Mice were immunized intraperitoneally with 50 µg of eMOD admixed to 1 µg of Bet v 1-alum (black bars; eMOD/sens) on days 0, 14 and 28, then intranasally challenged with 100 µg of birch pollen extract (BP) on three consecutive days one week after the last sensitization (days 35–37) and sacrificed on day 40. Control mice (white bars; sens) were immunized with Bet v 1-alum admixed to PBS. Serum was collected at day 40. Levels of Bet v 1-specific and eMOD-specific IgG1 (B; E); IgG2a (C; F), and eMOD-specific IgE (G) were measured by ELISA. Functional IgE was measured by Bet v 1-mediated β-hexosaminidase release from rat basophil leukemia cells (D). The dashed line indicates the background level of the assay. Results represent three combined experiments each with five mice per group. Data are expressed as mean ±SEM. ***p<0.001.

To study the effect of eMOD on allergen-mediated sensitization and intranasal challenge we used a mouse model of birch pollen allergy (Fig. 4 A). The application of eMOD together with Bet v 1 significantly reduced the development of Bet v 1-specific IgG1 as well as IgG2a isotypes in serum (Fig. 4 B, C). These data suggest that eMOD has a general effect on allergen-specific antibody production rather than an effect on the Th1/Th2 balance. Similarly, the application of eMOD during sensitization reduced the IgE-dependent basophil degranulation to Bet v1 compared with sensitized and challenged controls (Fig. 4 D). On the other hand, eMOD-specific antibody production in eMOD-treated, sensitized and challenged mice was significantly increased in comparison to sham-treated sensitized and challenged controls (Fig. 4 E–G).

### Application of eMOD during Sensitization with Bet v 1 Inhibits the Development of Allergic Airway Inflammation

**Figure 5:**
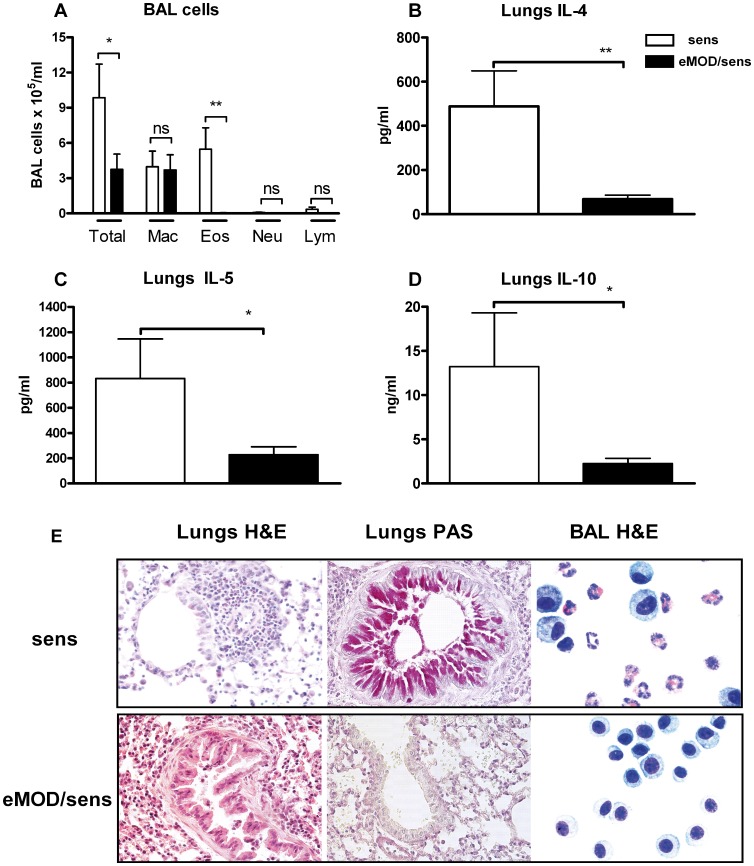
Co-application of eMOD at sensitization reduces the development of airway inflammation. Mice were immunized intraperitoneally with 50 µg of eMOD admixed to1 µg of Bet v 1-alum (black bars; eMOD/sens) on days 0, 14 and 28, then intranasally challenged with 100 µg of birch pollen extract (BP) on three consecutive days one week after the last sensitization (days 35–37) and sacrificed on day 40. Control mice (white bars; sens) were immunized with Bet v 1-alum admixed to PBS. Bronchoalveolar lavage fluid (BALF) and lung tissue for cell preparation and histology were collected at day 40. (A) The numbers of total and differential cells in BALF. Production of IL-4 (B), IL-5 (C) and IL-10 (D) in BP-re-stimulated lung cell cultures was assessed by ELISA. Results represent three combined experiments each with five mice per group. Data is expressed as mean ±SEM. *p<0.05; **p<0.01; Mac = macrophages; Eos = eosinophils, Neu = neutrophils; Lym = lymphocytes. (E) Representative haematoxylin and eosin (H&E) and periodic acid-Schiff (PAS) staining of paraformaldehyde-fixed lung tissue sections at 40×magnification. Representative BALF cytospins were stained with H&E and are shown at 100×magnification.

Three doses of eMOD during sensitization with Bet v 1 significantly suppressed the airway allergic inflammation measured by total cell numbers in the BALF after intranasal challenge with birch pollen extract (Fig. 5 A). The differential cell counting of cytospins revealed that the cellular infiltrates were reduced with eMOD administration, which was due primarily to the reduction of eosinophils (Fig. 5 A, E). H&E and PAS staining of formalin-fixed lung sections also showed a strong reduction of peribronchial and perivascular cellular infiltrations as well as reduced goblet cell hyperplasia and mucus production in mice treated with eMOD (Fig. 5 E). Administration of eMOD also suppressed production of IL-4, IL-5 as well as regulatory cytokine IL-10 in lung cells stimulated with birch pollen extract (Fig. 5 B–D).

### Systemic Allergen-specific Recall Responses are Reduced by eMOD Treatment

**Figure 6:**
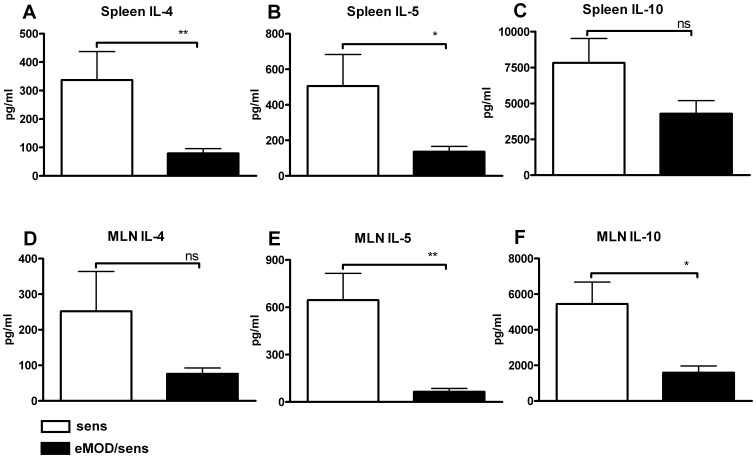
Co-application of eMOD at sensitization reduces allergen-specific recall responses. Mice were immunized intraperitoneally with 50 µg of eMOD admixed to 1 µg of Bet v 1 (black bars; eMOD/sens) on days 0, 14 and 28, then intranasally challenged with 100 µg of birch pollen extract (BP) on three consecutive days one week after the last sensitization (days 35–37). Control mice (white bars; sens) were immunized with Bet v 1 admixed to PBS only. On day 40, splenocytes and mesenteric lymph node (MLN) cells were stimulated with 50 µg/ml of BP extract for 72 hours. Levels of IL-4 (A, D), IL-5 (B, E) and IL-10 (C, F) were assessed by ELISA. Results are representative of at least two independent experiments each with five mice per group. Data is expressed as mean ±SEM. *p<0.05; **p<0.01.

To determine whether the suppressive effects of eMOD on airway inflammation were associated with changes in systemic cellular responses, we examined allergen-specific recall responses in splenocytes and MLN cells. We found that IL-4, IL-5 and IL-10 were all suppressed in both birch pollen extract stimulated spleen (Fig. 6 A–C) and MLN (Fig. 6 D–F) cultures derived from eMOD-treated mice in comparison to controls. Levels of IFN-γ were found not to differ between mice that had received eMOD and control mice (data not shown). These results provide additional evidence that the shift to Th1 responsiveness does not account for the down-regulatory effects of eMOD on Bet v 1-specific Th2 responses.

### The Potential of eMOD to Prevent the Development of Allergy is Mediated by Heat-stable Components

**Figure 7:**
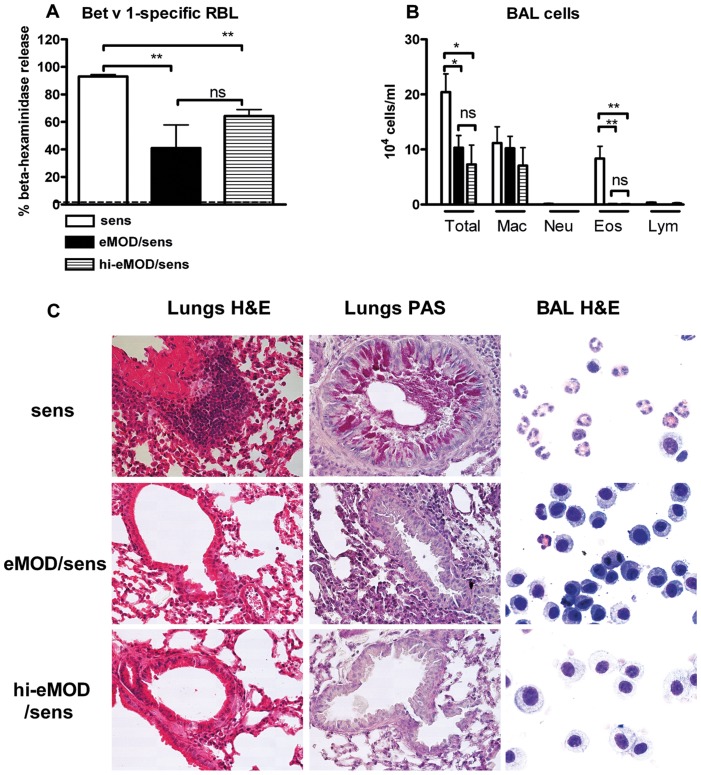
The suppressive effects of eMOD are heat-stable. Mice were immunized intraperitoneally with 1 µg of Bet v 1 admixed to 50 µg of eMOD (black bars; eMOD/sens) or heat-treated eMOD (striped bars; hi-eMOD/sens) on days 0, 14 and 28, then intranasally challenged with 100 µg of birch pollen extract (BP) on three consecutive days one week after the last sensitization (days 35–37). Control mice (white bars; sens) were immunized with Bet v 1 admixed to PBS. Serum, bronchoalveolar lavage fluid (BALF) and lung tissue for histology were collected at day 40. Functional IgE was measured by Bet v 1-mediated β-hexosaminidase release from rat basophil leukemia cells (A). The dashed line indicates the background level of the assay. (B) The numbers of total and differential cells in BALF. Results are representative of two experiments each with five mice per group. Data is expressed as mean ±SEM. **p<0.01; ns = not significant; Mac = macrophages; Eos = eosinophils, Neu = neutrophils; Lym = lymphocytes. (C) Representative haematoxylin and eosin (H&E) and periodic acid-Schiff (PAS) staining of paraformaldehyde-fixed lung tissue sections at 40×magnification. Representative BALF cytospins were stained with H&E and are shown at 100×magnification.

To elucidate whether heat-stable components of eMOD are responsible for the suppression of Bet v 1-specific responses, eMOD was heated at 96°C for 15 min to denaturate the protein components. As shown in Fig. 7, heat-treated eMOD could still reduce IgE-dependent basophil degranulation in response to Bet v1 (Fig. 7 A) and retained the ability to suppress allergen-induced airway eosinophilia (Fig. 7 B, C) and peribronchial and perivascular cellular infiltration and mucus production in lungs (Fig. 7 C).

### Co-administration of eMOD during Immunization Suppresses Humoral Responses to Thymus-Dependent but not to Thymus-independent Model Antigen

**Figure 8:**
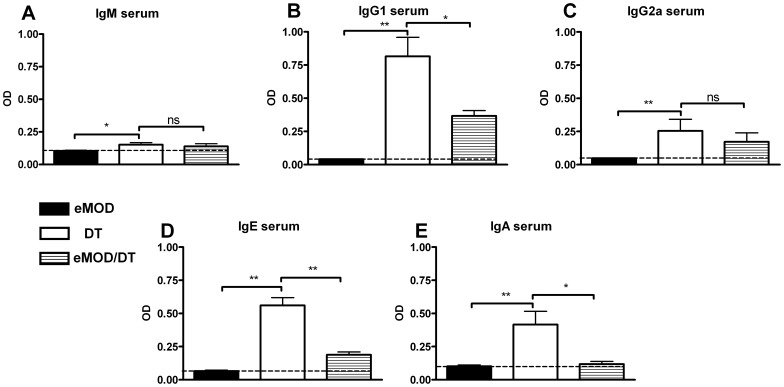
Co-application of eMOD suppresses humoral responses to thymus-dependent diphtheria toxoid antigen. Mice were immunized intraperitoneally with 50 µg of eMOD (dark bars; eMOD) or 10 µg of diphtheria toxoid-alum (white bars; DT) or 50 µg of eMOD admixed to 10 µg of diphtheria toxoid-alum (striped bars; eMOD/DT) on days 0 and 7. Sera were collected on day 21. Levels of DT-specific IgM (A), IgG1 (B), IgG2a (C), IgE (D), and IgA (E) were detected by ELISA. The dashed line indicates the background level of the assay. Results are representative of two experiments each with five mice per group and data are expressed as mean ±SEM. *p<0.05; **p<0.01; ns = not significant.

**Figure 9:**
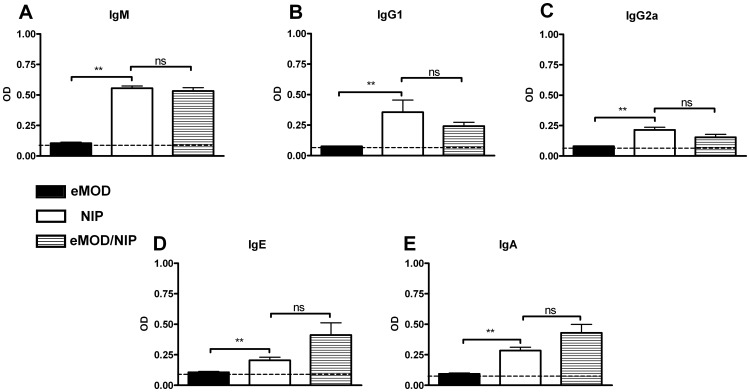
Co-application of eMOD does not interfere with humoral responses to thymus-independent NIP-Ficoll antigen. Mice were immunized intraperitoneally with 50 µg of eMOD (dark bars; eMOD) or 200 µg of NIP-Ficoll (white bars; NIP) or 50 µg of eMOD admixed to 200 µg of NIP-Ficoll (striped bars; eMOD/NIP) on days 0 and 7. Sera were collected on day 21. Levels of NIP-specific IgM (A), IgG1 (B), IgG2a (C), IgE (D), and IgA (E) were detected by ELISA. The dashed line indicates the background level of the assay. Results are representative of two experiments each with five mice per group and data are expressed as mean ±SEM. **p<0.01; ns = not significant.

To distinguish if the suppression of allergy is due to modulation of T helper cell or B cell function, we measured the impact of eMOD on responses to a thymus-dependent and -independent model antigens DT and NIP-Ficoll, respectively. Immunization with DT led to the production of DT-specific IgM, IgG (predominantly IgG1 but also IgG2a), IgE and IgA in serum, as expected (Fig. 8 A–E). When DT was co-administered with eMOD, anti-DT IgG1 (Fig. 8 B), IgE (Fig. 8 D) and IgA (Fig. 8 E) levels were significantly reduced. The responses to DT-specific IgM (Fig. 8 A) and IgG2a (Fig. 8 C) were not significantly influenced by eMOD. Next we investigated the impact of eMOD on humoral responses to thymus-independent model antigen NIP-Ficoll. Immunization of mice with NIP-Ficoll led to the production of specific IgM (Fig. 9 A), IgG1 (Fig. 9 B), IgG2a (Fig. 9 C), IgE (Fig. 9 D), and IgA (Fig. 9 E) antibodies in sera. These antibody responses were comparable between mice immunized with NIP-Ficoll and mice immunized with NIP-Ficoll admixed to eMOD (Fig. 9 A–E). Thus, immune responses to thymus independent antigen NIP-Ficoll which activates B cells and antibody production in the absence of T cell support were similar between NIP-Ficoll-immunized mice irrespective of the simultaneous administration of eMOD.

## Discussion

Our study has investigated the immune response to eMOD, i.e. a extract from male *O. dentatum,* and its interactions with the immune system in three different experimental settings: i) sensitization and aerosol challenge with birch pollen allergen (type I allergy); ii) immunization with the thymus-dependent model antigen DT; and iii) immunization with the thymus-independent model antigen NIP-Ficoll.

Type 2 immune responses, characterized by the induction of cytokines IL-4, IL-5, the antibody isotypes IgG1 and IgE, as well as expanded populations of eosinophils, are induced by and confer protection against helminth infections in both humans and animals [31]. Here we have shown that live infection is not a prerequisite for induction of type 2 immune response. Immunization of BALB/c mice with eMOD led to induction of both humoral and cellular type 2 responses. In serum, eMOD-specific IgG responses consisted predominantly of IgG1 (Th2-associated isotype). Similarly, eMOD stimulation of splenocytes and MLN cells derived from eMOD-immunized mice led to the production of IL-4 and IL-5 (Th2-associated cytokines). Mechanisms leading to the adaptive Th2 immunity are mainly dependent on early IL-4 production, as well as the essential participation of dendritic cells [32]. The induction and maintenance of Th2 responses in this new eMOD model would be well worth exploring in future work.

The regulatory cytokines IL-10 and TGF-β were also induced in eMOD-re-stimulated spleen and MLN cell cultures. Chronic helminth infections generally induce multiple regulatory pathways, involving regulatory T cells, B cells, dendritic cells, and macrophages, with IL-10 and TGF-β playing an important role [33], [34], [35]. Furthermore, responsiveness to vaccine antigens such as BCG was attenuated in helminth infected subjects with concomitant increase in levels of TGF-β [36].

Despite the fact that both helminth infections and external allergens induce similar immunological responses, epidemiological data indicate that infections with certain parasites, such as hookworms or schistosomes, can protect from allergic disorders [37]. Furthermore, excretory-secretory products from *H. polygyrus* can also suppress allergen-specific Th2 responses and pathology in the OVA-induced mouse model of type I allergy [23]. In our current study we investigated whether eMOD can protect against allergic disease in a model of clinically relevant birch pollen allergy. Co-administration of eMOD with recombinant allergen Bet v 1 significantly suppressed allergen specific immune responses. The fact that only low levels of Bet v 1-specific antibodies were detected in serum of eMOD/Bet v 1-treated mice in comparison to sensitized controls suggest that the presence of eMOD during the allergen-sensitization phase might interfere with the development of Bet v 1-specific responses, resulting in significantly reduced eosinophilia and mucus production after the intranasal challenge with birch pollen extract.

Helminth parasites produce enzymes with protease activity that facilitate their entry into the host [38]. Thus, proteases in eMOD could be responsible for the modification/digestion of Bet v 1 protein *in vivo*, preventing antigen-presenting cells, such as DC, from stimulating T cells. However, the fact that heat-treatment preserves the immunomodulatory effects of eMOD suggests that enzymatic effects do not play a major role in our model and suppression is likely to be mediated by heat-stable non-protein compounds.

We found that eMOD reduced production of both Th2-associated IgG1 and IgE as well as Th1-associated IgG2a to Bet v 1 in serum, suggesting that allergy inhibition was not achieved by immune deviation toward Th1 as previously suggested in other settings [39], [40]. This is of particular relevance since recent animal studies have shown that allergen-specific Th1 cells are recruited to the lung of sensitized and challenged mice, contributing to the development of severe airway inflammation and causing acute lung pathology [41], [42]. Thus, suppression of allergen-specific responses, rather than immune deviation toward allergen-specific Th1 responses might be the strategy of choice for the prevention of allergy.

Interestingly, eMOD suppresses Bet v 1-specific antibody production in serum but simultaneously induces production of eMOD-specific antibodies. Similarly, excretory/secretory products derived from *N. brasiliensis* (NES) were shown to induce NES-specific Th2 responses and at the same time inhibit the development of OVA-specific responses [22]. Again, these data suggest that eMOD prevents the development of allergy without evoking a general suppression of the immune system, indicating that this extract could be used for allergen-specific prevention strategies.

In view of the high amounts of IL-10 and TGF-β produced in eMOD-stimulated cell cultures, it can be expected that Treg (Foxp3+) cells will be increased by expansion or by *de novo* induction. There are numerous studies showing the importance of Treg cells in modulating host immune responses. In several animal infection models, helminth parasites such as *H. polygyrus* [43], *Litomosomoides sigmodontis* [44] or *Schistosoma mansoni* [45], induce regulatory responses via different regulatory cells, most prominently the CD4+CD25+Foxp3+ population [35]. Excretory/secretory products of *H. polygyrus* induced Foxp3 expression in T cells *in vitro* through the TGF-β pathway and are able to suppress airway allergy [46]. However, using Foxp3-eGFP mice we have shown recently that eMOD does not have a potential to expand and/or induce *de novo* T cells Foxp3 expression *in vitro* (data not shown). Similarly, Ilic *et al.* have shown that products derived from *T. spiralis* induce high levels of IL-10 but they do not impact on the existing Foxp3+ cell population or induce Foxp3+ cells *de novo* [47].

Regulatory cytokine IL-10 has been shown to play an important role in control of allergic airway disease by helminth infections or by helminth-derived products [48], [49]. Interestingly, levels of IL-10 were down-regulated similarly to IL-4 and IL-5 in birch pollen-stimulated splenocytes derived from eMOD-treated and sensitized mice. This observation is in agreement with our previous studies, in which prevention of allergy by application of probiotic bacteria was associated with reduced levels of IL-10 in allergen-re-stimulated splenocytes [50], [51].

Pre-existing helminth infections interfere not only with the development of allergy but also with the efficacy of different vaccines, including BCG [36], [52], [53], [54], [55], influenza [56], tetanus [57], [58] or malaria vaccine candidates [59], [60]. On the basis of these reports we investigated whether responses to both T cell-dependent (DT) and T cell-independent (NIP-Ficoll) model antigens can be modulated by eMOD. We show that immunization of mice with DT antigen admixed to eMOD resulted in significant reduction of DT-specific IgG1 and IgE. In contrast, eMOD has no impact on T cell-independent DT-specific IgM and NIP-Ficoll specific antibody production. NIP-Ficoll is a hapten coupled to polymeric molecule leading to B cell receptor cross-linking, partial B cell maturation, class switching and antibody production without the requirement of antigen presentation to T helper cells by antigen presenting cells (APC) [61], [62]. Thus, our data suggest that rather than impairing B cell responses, eMOD might interfere with T cell functions directly or through the effects on APCs, such as DC. Indeed, we have shown that stimulation of BM-DC leads to production of the regulatory milieu which might be responsible for suppression of T cell responses. Along these lines, Boitelle *et al*. report that helminth products interfered with the efficient expansion of OVA specific CD4 T cells *in vivo* [63] and infection with *S. ratti* directly suppresses antigen-specific proliferation of T helper cells [64]. Studies investigating the impact of eMOD on antigen uptake by dendritic cells and their antigen presentation to T cells are now under way.

Trujillo-Vargas *et al.* also showed that heat-treated and proteinase K-digested NES retained the ability to suppress OVA-induced airway eosinophilia, but did not retain the ability to suppress IgE responses, implicating both heat-stable and heat-labile suppressive compounds in NES [22]. In our study, heat-treatment of eMOD retained the protective effects against birch pollen allergy, as Bet v 1-specific RBL release, infiltration of eosinophils in BALF, and reduced peribronchial inflammatory infiltrate and mucus hypersecretion were markedly reduced in mice treated with both native and heat-treated eMOD in comparison to sensitized and challenged control animals. These data indicate that non-protein components, such as carbohydrates, may play an important role in allergy suppression. Previous data, based on lectin binding studies, indicate that *O. dentatum* displays stage-specific glycoconjugates [65], [66]. More recently, structural data on glycan decorations of *O. dentatum* structures were analyzed and galactosylated fucose epitopes have been identified [67]. We are currently carrying out experiments to identify the compounds in eMOD with immunomodulatory potential.

Taken together, we clearly established that eMOD inhibits the development of allergen-specific immune responses including antibody production, cellular recall responses, and airway eosinophilia in a mouse model of allergy. Furthermore, the allergy-protective effects of eMOD are mediated by heat-stable compounds which might interfere with antigen presenting cells or T cell function. Understanding of the molecular cross-talk between chronic helminth infections and the host immune system as well as identification of parasite-derived molecules involved in immunomodulation could now pave the way to novel prophylactic/therapeutic strategies for immune dysfunctions such as allergies and autoimmunity.
